# Maximal Eccentric Hamstrings Strength in Competitive Alpine Skiers: Cross-Sectional Observations From Youth to Elite Level

**DOI:** 10.3389/fphys.2019.00088

**Published:** 2019-02-18

**Authors:** Martino V. Franchi, Lynn Ellenberger, Marie Javet, Björn Bruhin, Michael Romann, Walter O. Frey, Jörg Spörri

**Affiliations:** ^1^Sports Medical Research Group, Department of Orthopaedics, Balgrist University Hospital, University of Zurich, Zurich, Switzerland; ^2^Laboratory for Muscle Plasticity, Department of Orthopaedics, Balgrist University Hospital, University of Zurich, Zurich, Switzerland; ^3^Section for Elite Sport, Swiss Federal Institute of Sport Magglingen, Magglingen, Switzerland; ^4^Swiss-Ski, Muri bei Bern, Switzerland; ^5^Balgrist Move>Med, Department of Orthopaedics, Balgrist University Hospital, University of Zurich, Zurich, Switzerland

**Keywords:** conditioning, physical fitness, neuromuscular performance, testing, biological maturity status, athletes, injury prevention, alpine ski racing

## Abstract

Competitive alpine skiers are subject to substantial risks of injury, especially concerning the anterior cruciate ligament (ACL). During “landing back weighted” episodes, hamstrings may partially counteract the anterior shear force acting on the tibia by eccentrically resisting the boot-induced drawer of the tibia relative to the femur. The aim of the present study was to provide novel descriptive data and sport-specific reference values on maximal eccentric hamstrings strength (MEHS) in competitive alpine skiers from youth to elite level, and to explore potential relationships with sex, age and biological maturation. 170 competitive alpine skiers were investigated: 139 youth athletes (51 females, 88 males; age: 13.8 ± 0.59 years) and 31 elite athletes (19 females, 12 males; age: 21.7 ± 2.8 years). MEHS was assessed by the (Vald Performance, Newstead, Australia). U15 female skiers presented lower MEHS compared to female elite skiers for both limbs (*R* = 210 ± 44 N vs. 340 ± 48 N, respectively, *p* < 0.001, and *L* = 207 ± 46 N vs. 303 ± 35 N, respectively, *p* < 0.001). Similarly, lower MEHS was observed in U15 male skiers compared to male elite skiers for both limbs (*R* = 259 ± 51 N vs. 486 ± 62 N, respectively, *p* < 0.001, and *L* = 258 ± 57 N vs. 427 ± 54 N, respectively, *p* < 0.001). Correlations between MEHS and chronological age were modestly significant only for the U15 group (*r* = 0.37 and *p* < 0.001). When the correlations for the U15 group were performed between MHES and maturity offset (obtained from the calculation of biological age, i.e., age at peak height velocity), statistical significance was reached by all the correlations run for 3 variables (Males < 0: *r* = 0.59, *p* < 0.0001; Males > 0: *r* = 0.70, *p* < 0.0001; and Females > 0: *r* = 0.46, *p* < 0.0001, start of maturity offset = 0). This cross-sectional description of MEHS in alpine skiers from youth to elite level highlights the importance of biological maturation for MEHS values in youth athletes and presents novel data that may offer insights into new approaches for injury prevention.

## Introduction

Competitive alpine skiers are known to be subject to substantial risks of injury (Spörri et al., 2017). Although the rates for some injuries have been recently reported to show a decline as stated by Färber et al. (2018), the possibility for skiers to sustain an anterior cruciate ligament (ACL) injury during their sportive career is still very high (Pujol et al., 2007; Flørenes et al., 2009, 2012; Westin et al., 2012, 2018; Bere et al., 2013a; Stenroos and Handolin, 2014; Haaland et al., 2016; Müller et al., 2017b). Most of the ACL-injuries occur while the skier is turning or landing from a jump (i.e., before or without falling) (Bere et al., 2011, 2014). Typical ACL-injury mechanisms include excessive knee joint compression, knee valgus and internal rotation, or a boot-induced anterior drawer of the tibia relative to the femur (Bere et al., 2011, 2013b; Jordan et al., 2017; Spörri et al., 2017).

Physical aspects of the athlete have been suggested to be among the top 5 key injury risk factors in alpine ski racing (Spörri et al., 2012) and fitness parameters have been shown to be associated with injury risk (Raschner et al., 2012; Müller et al., 2017a). During typical ACL-injury mechanisms, such as the “landing back weighted” mechanism, hamstring muscles may act as an ACL-synergist by producing a posteriorly directed shear force to the tibia (i.e., by eccentrically resisting the boot-induced anterior drawer of the tibia relative to the femur while landing).

Considering that both quadriceps and hamstrings muscle groups are significantly activated during jump landings (Färber et al., 2018), it is reasonable to enquire whether enhanced co-activation of such muscle groups contributes to prevention strategies (Oberhofer et al., 2017). However, previous research and opinion targeting quadriceps functional features and ACL-injuries has been controversial, as Färber et al. (2018) pointed out. Instead, hamstrings strength capacity may be of importance for many typical injury situations (e.g., jump landings or backward falls) (Read and Herzog, 1992; Herzog and Read, 1993; Gerritsen et al., 1996; DeMorat et al., 2004; Koyanagi et al., 2006; Semadeni and Schmitt, 2009; Bere et al., 2011, 2014; Yeow et al., 2011; Heinrich et al., 2018). In fact, if hamstrings are pre-activated fast and high enough (Färber et al., 2018), tibial anterior translation relative to the femur might be reduced, consequently diminishing the risk of ACL-injury.

Eccentric muscle actions are an inherent part of skiing (Berg et al., 1995; Kröll et al., 2015a,b), and specifically, sufficient eccentric hamstrings strength is considered to be important for ACL-injury prevention in skiers (Jordan et al., 2017; Spörri et al., 2017) and in athletes in general (Bourne et al., 2018). However, to date, there is no study that comprehensively investigated maximal eccentric hamstrings strength (MEHS) neither in youth nor in elite competitive alpine skiers. Thus, although it could be of significant interest for injury prevention strategies, to our knowledge, there is no presence in literature of any observations regarding relationships between sex, sportive level, chronological age/ biological maturation and MEHS in competitive alpine skiers. Gaining further information on such parameters could help to identify potential new stratagems for ACL-injury prevention in youth and elite skiers and to better understand how to implement MEHS related prevention strategies effectively.

Accordingly, the sub-goals of the present study were: (1) to screen two distinct populations of competitive alpine skiers (including youth athletes and elite athletes) by assessing MEHS during Nordic Hamstrings Exercise (NHE), which has extensively been used in different sports such as Australian football, rugby, soccer and sprinting (Opar et al., 2013; Timmins et al., 2016); (2) to conduct a cross-sectional observation (from youth to elite level) on various relationships between sex, sportive level, age, biological maturation and MEHS. The overall aim of the present study was to provide novel descriptive data and reference values on MEHS in competitive alpine skiers, which could be of strategical interest for future novel injury prevention approaches starting from youth competitive level and age.

## Materials and Methods

### Participants and Study Design

In total 170 competitive alpine skiers participated in the study: 139 U15 youth athletes (51 females, 88 males; mean age: 13.8 ± 0.6 years; range: 12.9 – 14.9 years) and 31 adult athletes (19 females, 12 males; mean age: 21.7 ± 2.8 years; range: 17.0 – 28.9 years). Table 1 provides detailed anthropometric data separated by gender and groups of youth and adult elite skiers. Measurements were completed during the preseason (October 2017–November 2017) for youth elite alpine skiers and during off-season (May 2018–June 2018) for national level ski racers. This study was carried out in accordance with the recommendations of the institutional review board and local ethic committee with written informed consent from all subjects in accordance with the Declaration of Helsinki. Study approval was granted by the institutional review board and local ethic committee (KEK-ZH-NR: 2017-01395).

**Table 1:** Anthropometric data for male and female athletes separated by groups.

	U15 athletes		Elite athletes	

	Female	Male	Female	male
	
	Mean (±SD) (min-max)	Mean (±SD) (min–max)	Mean (±SD) (min-max)	Mean (±SD) (min-max)
**Age [y]**	13.7 ± 0.6 (12.5–14.9)	13.9 ± 0.5 (12.9–14.8)	21.3 ± 2.7 (17–26.3)	22.4 ± 2.9 (18.3–28.9)
**Body height [cm]**	159.7 ± 6.3 (143–171.5)	161.4 ± 8.4 (145–185)	166.4 ± 5.7 (155–180)	176.3 ± 6.7 (166–189)
**Body weight[kg]**	48.4 ± 7.6 (35–66.5)	49.4 ± 10.3 (30–81)	65.5 ± 5.9 (50–82)	80.8 ± 6.2 (71–103)
**BMI [kg/m^2^]**	18.9 ± 1.2 (14.4–25.9)	18.9 ± 1.2 (13–24.7)	23.6 ± 1.8 (20–26.3)	25.9 ± 1.3 (23.4–29)

*SD, standard deviation.*

### Maximal Eccentric Hamstring Strength During NHE

The maximal eccentric hamstring strength was assessed by using a NHE measurement device (Vald Performance, Newstead, Australia); its reliability and application on athlete populations is reported in several previous studies (Bourne et al., 2015; Opar et al., 2015; Timmins et al., 2016). Briefly, athletes knee on a padded board of the Norbord device with their ankles fixed by braces right above the lateral malleoli. The ankle braces contain integrated uniaxial load cells which are affixed to a pivot in order to ensure a constant force measurement through the longitudinal axis of the load cell. Directly prior to the measurement an investigator demonstrated the NHE to each athlete. The following verbal instructions were provided as previously described (Bourne et al., 2015; Opar et al., 2015): gradually lean forward at the slowest possible speed; maximally resist this movement with both legs; keep trunk and hips in a neutral position throughout the movement; hold hands crossed above the chest. A repetition was completed if the resulting forces overcame the athlete’s resistance and pressurized a catch of the movement with the hands on the ground. All participants performed one set of three repetitions of NHE (5–10 s of rest between repetitions), whereby they were verbally encouraged to secure maximal exertion. Based on the previously described instructions a trial was considered valid if it demonstrated a constant increase of force progression culminating in a pronounced force peak, followed by a rapid decline. The best left and right maximum values of the three repetitions were used for further data analysis. The limbs asymmetry during MEHS production during NHE test was calculated as the difference between stronger and weaker leg expressed as percentage.

### Biological Age and Maturity Offset Calculation

The biological age was calculated based on a formula by Mirwald et al. (2002) which provides a non-invasive and previously validated method to predict the age at peak height velocity (APHV) (Malina et al., 2007; Sherar et al., 2007) and moreover was validated for youth competitive alpine skiers (Müller et al., 2015). The gender-specific equations use anthropometric measures of body mass (0.1 kg, *Seca, Hamburg, Germany*), body height and sitting height (0.5 cm, determined by measuring tape), as well as chronological age at the time of measurement and sub-ischial leg length as the difference between body height and sitting height. Based on the collected data the prediction of an individual maturity offset is enabled, which marks a point in time before or after peak height velocity (PHV). The estimated APHV is given by subtracting the maturity offset from the actual chronological age (Mirwald et al., 2002).

### Statistical Analysis

Data were reported as mean ± SD. Differences between groups were statistically analyzed for MEHS values using an unpaired Student’s *t*-test. Correlations between sex, chronological age, biological age and MEHS and were tested by the Pearson’s product moment correlation coefficient (*r*) and coefficient of determination (*r^2^*). The level of significance was set at *p* < 0.05.

## Results

### Maximal Eccentric Hamstring Strength During NHE

**FIGURE 1:**
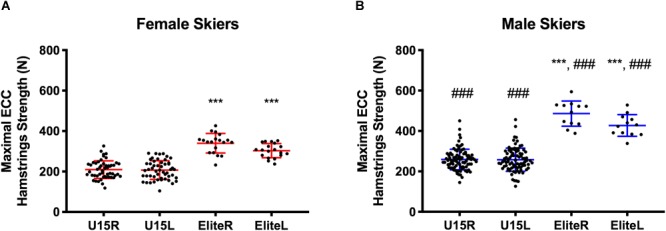
Maximal eccentric hamstrings strength of right and left limbs for U15 and Elite athletes. ^∗∗∗^ = significantly different between Elite and U15 group of the same sex, *P* < 0.001; ### = significantly different between the same age/sportive level but of different sex, *P* < 0.001. **(A)** female, **(B)** male.

A total of 170 competitive alpine skiers performed a maximal NHE-test. A comprehensive snapshot of the differences in strength between females and males for different limbs and at different age/sportive level is presented in Figure 1A,B. Figure 1A shows that U15 female skiers (*n* = 51) presented a significantly lower eccentric hamstrings strength compared to female elite skiers (*n* = 19) for both right and left limbs (*R* = 210 ± 44 N vs. 340 ± 48 N, respectively, *p* < 0.001, and *L* = 207 ± 46 N vs. 303 ± 35 N, respectively, *p* < 0.001). Similarly, Figure 1B shows that a significantly lower eccentric hamstrings strength was observed in U15 male skiers (*N* = 88) compared to male elite skiers (*n* = 12) for both limbs (*R* = 259 ± 51 N vs. 486 ± 62 N, respectively, *p* < 0.001, and *L* = 258 ± 57.2 vs. 427 ± 54 N, respectively, *p* < 0.001). Male skiers always presented significantly higher values of eccentric hamstrings strength irrespective of limb, age, or sportive level compared to female skiers (*p* < 0.001).

### Between Limbs Imbalance (Asymmetry) During NHE

**FIGURE 2:**
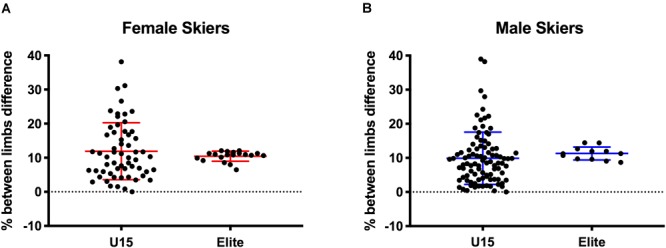
Limbs asymmetry of MEHS production during NHE for female and male skiers of both sportive level groups. **(A)** female, **(B)** male.

Data for asymmetry of force production between right and left limb during NHE (difference between stronger and weaker leg expressed as percentage), are presented in Figure 2A,B for U15 and elite skiers, females and males, respectively. U15 female skiers presented similar values of asymmetry for eccentric hamstrings strength production compared to female elite skiers (11.91 ± 8.3% vs. 10.46 ± 1.47%, *p* = 0.45); in a very similar fashion, U15 male skiers showed no significant differences of asymmetry for eccentric hamstrings strength production when compared to male elite skiers (9.88 ± 7.67% vs. 11.31 ± 1.89%, *p* = 0.52). However, it is worth mentioning that compared to elite skiers, U15 skiers showed a higher variance in the asymmetry values observed.

### Associations Between Maximal Eccentric Hamstrings Strength, Sex, Age and Maturity Offset

**FIGURE 3:**
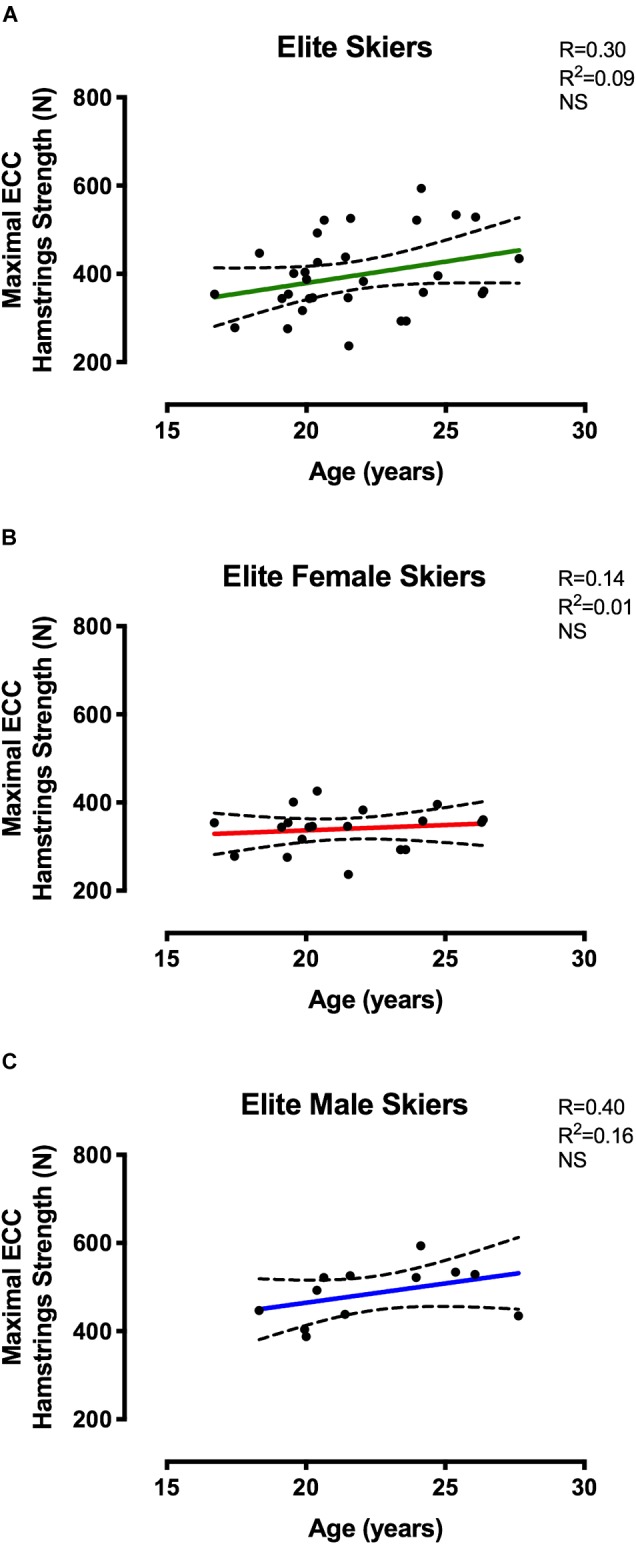
Correlations between MEHS and chronological age for **(A)** Elite Swiss Teams skiers irrespective of sex, **(B)** Elite female skiers, and **(C)** Elite male skiers.

**FIGURE 4:**
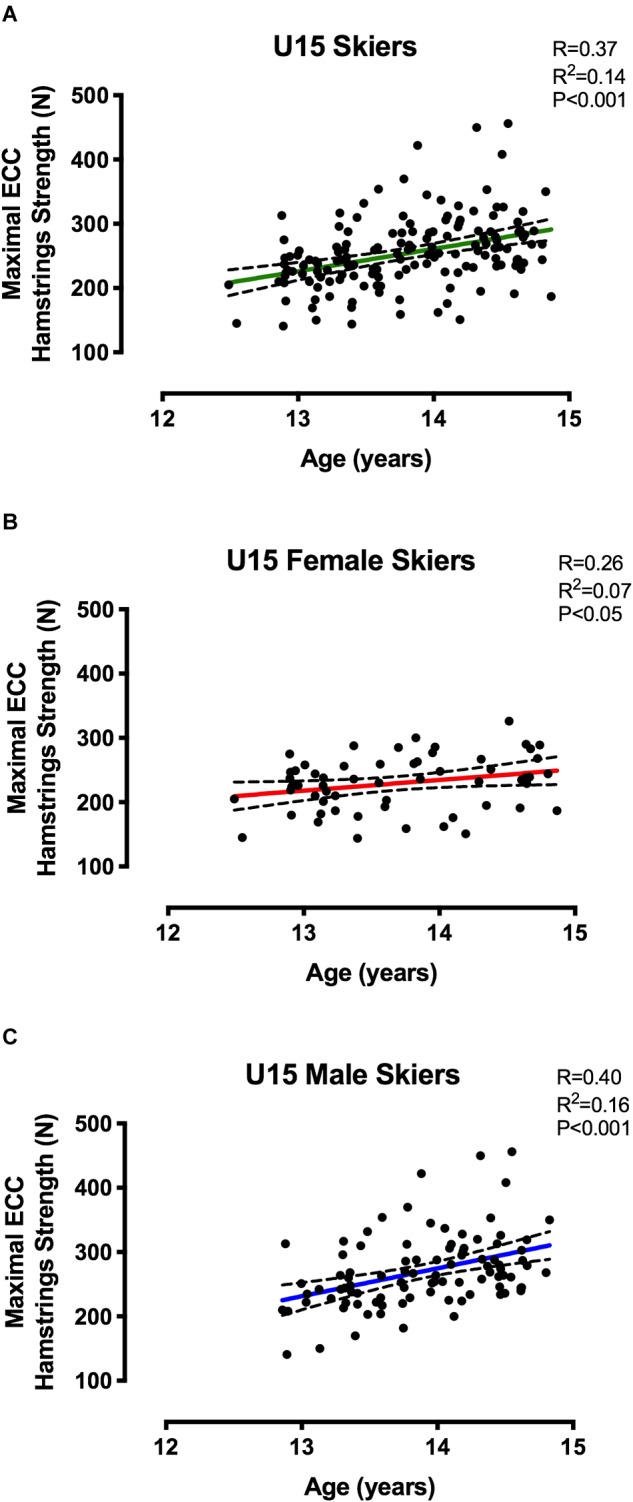
Correlations between MEHS and chronological age for **(A)** U15 skiers irrespective of sex, **(B)** U15 female skiers, and **(C)** U15 male skiers.

Correlations between MEHS and chronological age are presented in Figure 3 for the elite skiers and in Figure 4 for the U15 skiers, showed as grouped data (Figure 3A, 4A), as well as the data obtained when accounting for sex differences (Figure 3B,C, 4B,C). Pearsons’ *r* and *r*^2^-values, together with statistical significance, are shown in Figure 3, 4.

**FIGURE 5:**
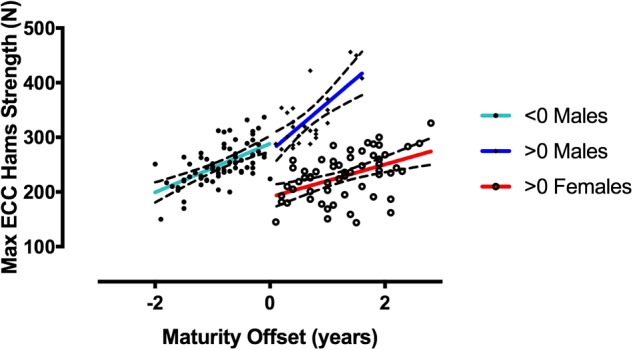
Correlations between MEHS and maturity offset is represented by 0 value. Males < 0: *r* = 0.59, *r*^2^ = 0.35, *p* < 0.0001; Males > 0: *r* = 0.70, *r*^2^ = 0.49, *p* < 0.0001; and Females > 0: *r* = 0.46, *r*^2^ = 0.22, *p* < 0.0001.

Pearson’s correlation between MEHS and chronological age did not reach any statistical significance in the elite group, neither for the grouped data (*r* = 0.30, *r*^2^ = 0.09, *p* = 0.1) nor for female and male groups (*r* = 0.14, *r*^2^ = 0.01, *p* = 0.56; *r* = 0.40, *r*^2^ = 0.16, *p* = 0.18). Conversely, the correlations for the U15 group were observed to be statistically significant when the data were grouped (*r* = 0.37, *r*^2^ = 0.14, *p* < 0.001) and when the data were expressed by sex (Females: *r* = 0.26, *r*^2^ = 0.07, *p* < 0.05 and Males: *r* = 0.40, *r*^2^ = 0.16, *p* < 0.001). When the correlations for the U15 group were performed between MEHS and maturity offset (Figure 5), statistical significance was reached by all the correlations run for 3 variables (Males < 0: *r* = 0.59, *r*^2^ = 0.35, *p* < 0.0001; Males > 0: *r* = 0.70, *r*^2^ = 0.49, *p* < 0.0001; and Females > 0: *r* = 0.46, *r*^2^ = 0.22, *p* < 0.0001, where 0 represents the start of maturity offset).

### Body Weight Normalized Maximal Eccentric Hamstrings Strength vs. Maturity Offset

**FIGURE 6:**
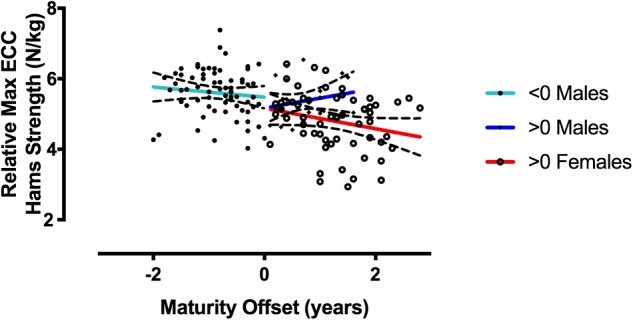
Correlations between relative MEHS (N/kg) and maturity offset is represented by 0 value. No significant correlations observed.

In the group of U15 skiers, correlations between MEHS and maturity offset disappear when a normalization with body weight is performed (Figure 6) (Males < 0: *r* = -0.1, *r*^2^ = 0.01, *p* = 0.38; Males > 0: *r* = 0.20, *r*^2^ = 0.04, *p* = 0.32; and Females > 0: *r* = -0.23, *r*^2^ = 0.05, *p* = 0.08, where 0 represents the start of maturity offset). Accordingly, a relative MEHS value-based ranking among the skiers is apparently different than an absolute value-based ranking (Figure 5).

## Discussion

The present investigation aimed to provide a cross-sectional observation of MEHS values in 170 competitive skiers (139 U15 athletes vs. 31 elite athletes). The main findings were the following: (1) greater MEHS during NHE was observed by the elite skiers compared to the U15 group and greater strength was developed by male compared to female skiers for both groups. (2) While no correlation was found between strength and chronological age in elite skiers, a weak to moderate association was found in the U15 group (*r* = 0.37 and *r*^2^ = 0.14). However, when strength was correlated to maturation offset for the latter group, this association showed moderate to strong linear relationships in a gender dependent manner (Males < 0: *r* = 0.59, *r*^2^ = 0.35; Males > 0: *r* = 0.70, *r*^2^ = 0.49; and Females > 0: *r* = 0.46, *r*^2^ = 0.22; where 0 represents the start of maturity offset). (3) In the U15 athletes, a body weight normalization of the MEHS values removes any relations to maturity offset.

### Toward Alpine Skiing-Specific Reference Values of Maximal Eccentric Hamstrings Strength and Between-Limb Imbalance

The individual and average absolute values for MEHS for both limbs are presented in Figure 1. The mean value for the male elite skiers was 486 ± 62.39 N for the right leg and 427.1 ± 53.62 N for the left one: compared to other previous studies, these values are considerably above the average found for elite Australian footballers (which did not sustain hamstrings injuries, average of left and right limbs = 301 ± 84 N, *n* = 159) (Opar et al., 2015), and for elite Rugby Union players (which did not sustain hamstrings injuries, average of left and right limbs = 367.7 ± 85 N, *n* = 158) (Bourne et al., 2015), and for football (soccer) players (which did not sustain hamstrings injuries, average of left and right limbs = 309.5 ± 73.4 N, *n* = 105) (Timmins et al., 2016). It must be stated that these higher force values may be also due to the high force production in the antagonists of the hamstring muscles (i.e., the knee extensors), which is typical for alpine ski racing and, therefore (Berg et al., 1995; Berg and Eiken, 1999), a priority in the conditioning of competitive alpine skiers. Lower values were observed for elite female athletes and U15 male and female skiers: however, to the best of our knowledge, no previous reports of MEHS (measured during NHE) were found comparable to these cohorts. The present study aimed purposely to provide the literature in sports medicine research with new sport-specific reference data on different cohorts of elite competitive skiers, also for multiple comparisons with different athletic populations.

Average values of between limb imbalance (asymmetry) for force developed during NHE was similar between the U15 and Elite groups and between sexes (*F* = 11.91 ± 8.3% vs. 10.46 ± 1.47%, respectively; *M* = 9.88 ± 7.67% vs. 11.31 ± 1.89%, respectively) (Figure 2). The present values are very similar to the ones previously showed for limbs in which no hamstrings injury had occurred in Australian footballers and rugby union players (Bourne et al., 2015; Opar et al., 2015). Moreover, it is worth highlighting that the individual values for U15 groups showed a great variability of between limb imbalance, possibly suggesting that U15 coordination strategies during NHE are not strongly consolidated.

### The Unexplored Role of Maximal Eccentric Hamstring Strength and Between-Limb Imbalance for the Risk of ACL Injuries in Alpine Ski Racing

An indirect (i.e., etiology/injury mechanism-based) justification of why MEHS may be of importance for the purpose of ACL injury prevention in alpine ski racing can be found in the following theoretical considerations. During typical ACL-injury mechanisms, such as the “landing back weighted” mechanism, hamstring muscles may functionally counteract the anterior shear force acting on the tibia (i.e., by eccentrically resisting the boot-induced anterior drawer of the tibia relative to the femur while landing). This hypothesis is further supported by the simulation study findings of (Semadeni and Schmitt, 2009), the fact that hamstring muscle activation levels can be voluntarily increased during jump landing (Färber et al., 2018), as well as the evidence of multimodal neuromuscular injury prevention programs (and NHE in particular) being effective in the reduction of the risk of ACL injury in sports other than alpine ski racing (Petushek et al., 2018). Moreover, higher values of between-limb imbalance (i.e., ranging from 21.2 to a 13.1% between start and the end of pre-season) have been associated with a risk of hamstring injury in rugby (Bourne et al., 2015). At the same time, it is still unclear if MEHS and/or between-limb imbalance could represent a risk factor for (side-dependent) ACL-related injuries in the sport of alpine ski racing. Accordingly, in a next step, longitudinal (i.e., epidemiology, etiology and/or intervention-related) studies are needed to verify the hypothesis of a direct association between MEHS, between limb imbalance and the risk of ACL injuries in the sport of alpine ski racing.

However, irrespective of these future aims, it is important to know about sport-specific reference values, potential age/maturity related influences and asymmetry problems in the corresponding populations, as being explored in the current study. Such information is essential for the interpretation of forthcoming longitudinal studies, and to better understand how to implement MEHS related prevention strategies effectively.

### The Associations of Sex, Sportive Level, Chronological Age and Biological Maturation With Maximal Eccentric Hamstrings Strength

It was no surprise that elite skiers (ranging from 17 to 28 years old) showed greater MEHS compared to the younger cohort (ranging from 12 to 15 years old): however, we further aimed to clarify if such discrepancy was just due to the age difference or to the fact that the two groups belonged to two distinct sportive levels (Figure 3–5). MEHS was not associated to chronological age in elite skiers: this may indicate that the individual differences in strength between elite athletes were potentially more due to training-related than just temporal factors.

Conversely, U15 athletes showed significant correlations between MEHS and chronological age when subjects were grouped irrespectively of gender (*r* = 0.37) and when divided for sex (*r* = 0.26 for female skiers and *r* = 0.40 for male skiers). However, these correlations presented very low *r*^2^-values (0.14, 0.07, and 0.16, respectively), thus explaining, in the best of the cases (i.e., for male skiers), only up to 16% of the variability of strength vs. age. Accordingly, we decided to investigate the relationship of eccentric hamstrings strength for female and male skiers in function of maturity offset (obtained from the calculation of biological age, i.e., age at peak height velocity). Interestingly, these relationships resulted in higher *r*^2^-values (Figure 5): males presented the most significant relationships before (*r*^2^ = 0.35) and after (*r*^2^ = 0.49) peak height velocity, while females showed and *r*^2^-value of 0.22 after peak height velocity. It must be specified that all of the female subjects have already reached their peak height velocity, so we could not present any relationship between maximal eccentric strength and maturity offset in the months/years before 0 value (i.e., the actual maturity offset). This is due to the fact that in this and other studies females reached their peak height velocity earlier than males (possibly around 11–12 years old) (Müller et al., 2017a,b).

The male subjects who already reached their peak height velocity were the ones that presented the higher absolute values for MEHS: while this was an expected finding, it is worth highlighting that few athletes within a year before PHV showed similar, if not greater, values of MEHS compared to other skiers which already passed PHV. In our opinion, these observations could be potentially regarded as selection criteria for either successive injury risk or general athletic performance, as young skiers that present such values of MEHS before complete maturation may start from a better overall condition compared to their peers, in a future perspective.

### Future Perspectives

The aforementioned correlations between biological maturation and MEHS suggest that in younger cohorts is important to consider if an athlete has already reached her/his peak height velocity point, in order to better interpret force values in key of injury prevention and performance. In fact, the lower force values observed in the U15 male skiers’ group were identified for athletes that were between 2 and 1 years before peak height velocity: if one would have considered just strength and/or chronological age for such subjects, potential misinterpretations could have been made for conclusions on risks of injury and/or selection criteria for high performance paths. One potential solution to better address the influence of biological maturation on MEHS in future might be found in a normalization with body weight (i.e., relative strength, N/kg). On the current study population such a normalization removed any relations between maturity offset and MEHS (Figure 6). However, for providing a deeper understanding of the long-term development of MEHS during the sportive career and/or over the entire life-span, future research should address the topic by the use of longitudinal study designs.

## Conclusion

This study aimed on a cross-sectional description of MEHS in competitive alpine skiers from youth to elite level. It may provide reference values and background knowledge for the interpretation/implementation of future ACL-injury prevention and athletic conditioning studies/interventions in the sport of alpine ski racing. Moreover, it highlighted the importance of considering biological maturation for meaningful interpretations of force values of youth athletes that are close to their growth spurts. Future investigations of MEHS in the context of ACL-injury prevention and/or athletic conditioning should focus on longitudinal observations of the same athletes during their sportive career. More integrative approaches should be implemented, such as combining muscle function testing with ultrasound-based assessment of hamstrings muscle mechanical behavior, its architectural adaptations to longitudinal training and the investigation of potential underlying molecular mechanisms.

## Author Contributions

JS and WF conceptualized the study. JS, LE, and MJ conducted the data collection. MF and LE contributed to the analysis and interpretation of the data. MF, LE, and JS drafted the manuscript. All other authors revised it critically, and approved the final version and agreed to be accountable for all aspects of this work.

## Conflict of Interest Statement

MJ and BB were professionally affiliated with Swiss-Ski. The remaining authors declare that the research was conducted in the absence of any commercial or financial relationships that could be construed as a potential conflict of interest.
